# The effect of omeprazole on the development of experimental autoimmune encephalomyelitis in C57BL/6J and SJL/J mice

**DOI:** 10.1186/1756-0500-7-605

**Published:** 2014-09-04

**Authors:** Scott A Sands, Sheila Tsau, Thomas M Yankee, Brooks L Parker, Aaron C Ericsson, Steven M LeVine

**Affiliations:** Department of Molecular and Integrative Physiology, University of Kansas Medical Center, 3901 Rainbow Blvd, Kansas City, 66160 KS USA; Department of Microbiology, Molecular Genetics and Immunology, University of Kansas Medical Center, 3901 Rainbow Blvd, 66160 Kansas City, KS USA; Department of Veterinary Pathobiology, Mutant Mouse Regional Resource Center, University of Missouri, 4011 Discovery Drive, 65201 Columbia, MO USA

**Keywords:** *Akkermansia muciniphila*, *Bacteroidales*, *Coprococcus*, Multiple sclerosis, Proton pump inhibitor, T cells

## Abstract

**Background:**

Gastric disturbances such as dyspepsia are routinely encountered by multiple sclerosis (MS) patients, and these conditions are often treated with gastric acid suppressors such as proton pump inhibitors, histamine H2 receptor antagonists, or antacids. The proton pump inhibitor omeprazole can alter the gut flora and immune responses, both of which can influence the course of experimental autoimmune encephalomyelitis (EAE), an animal model of MS. The objective of the current study was to examine the effect of omeprazole treatment on the development of EAE. Bacterial microbiome analysis of mouse fecal pellets was determined in C57BL/6J EAE mice chronically treated with omeprazole, and spleen immune cell content, clinical scores, weight, rotarod latency, and histopathology were used as outcome measures in C57BL/6J and SJL/J mice with EAE.

**Results:**

Omeprazole treatment resulted in decreases in *Akkermansia muciniphila* and *Coprococcus* sp. and an increase in unidentified bacteria in the family S24-7 (order *Bacteroidales*) in C57BL/6J mice with EAE. Omeprazole did not alter spleen immune cell content compared to vehicle in EAE mice, but differences independent of treatment were observed in subsets of T cells between early and advanced disease in C57BL/6J mice as well as between the two strains of mice at an advanced disease stage. Omeprazole caused no difference in clinical scores in either strain, but significantly lowered weight gain compared to vehicle in the C57BL/6J mice with EAE. Omeprazole also did not alter rotarod behavior or hindbrain inflammatory cell infiltration compared to vehicle in both strains of mice with EAE. Rotarod latency did reveal a negative correlation with clinical scores during active disease in both mouse strains, but not during clinical remission in SJL/J mice, suggesting that rotarod can detect disability not reflected in the clinical scores.

**Conclusions:**

Despite alterations in the gut microbiota and weight gain in the C57BL/6J EAE model, omeprazole had no effect on multiple measures of disease activity in C57BL/6J and SJL/J mice with EAE, supporting the notion that omeprazole does not substantially influence disease activity in MS patients.

**Electronic supplementary material:**

The online version of this article (doi:10.1186/1756-0500-7-605) contains supplementary material, which is available to authorized users.

## Background

Gastric disturbances are commonly encountered in multiple sclerosis (MS) patients, with ~30% reporting problems with dyspepsia [1]. Many of these disturbances are treated with gastric acid suppressors such as proton pump inhibitors (PPI), histamine H2 receptor antagonists, or antacids. These gastric acid suppressors are widely used, available over the counter, and generally considered safe; however, there have been concerns about susceptibility to small intestinal bacterial overgrowth [2] and enteric infections [3]. Since the gut microflora has been shown to influence the disease course in experimental autoimmune encephalomyelitis (EAE) [4–6], an animal model of MS, a PPI could theoretically impact disease progression by altering the microbiota. In addition, PPIs can directly affect the immune response [7], raising the possibility of a second mechanism of action by PPIs on disease progression. The objective of this study was to evaluate the effect of omeprazole treatment on the development of EAE in the C57BL/6J model of progressive MS and the SJL/J model of relapsing-remitting MS.

## Methods

### Animal handling and housing

All studies involving the use of animals were approved by the Institutional Animal Care and Use Committee of the University of Kansas Medical Center. Female C57BL/6J and female SJL/J mice (Jackson Laboratory, Bar Harbor, ME) were used for EAE induction. Animals were maintained on standard rodent chow (8604 Teklad rodent diet, Harlan, Indianapolis, IN), had water available *ad libitum*, and were maintained on a 12/12 h light/dark cycle. C57BL/6J mice were given 31M Nutrigel (ClearH2O, Portland, ME) supplementation as mice started to develop advanced disease, with all mice on Nutrigel by Day 27 post-encephalitogen injection. SJL/J mice that developed advanced disease, and their cage mates, received supplementation with DietGel Recovery Nutrigel (ClearH2O).

### EAE induction - C57BL/6J mice

EAE was induced in ~5 week old C57BL/6J female mice (Jackson Laboratory). Two studies were conducted; the first study lasted until Day 18 post-encephalitogen, the point at which disease was first detected (early clinical), while the second study lasted until Day 46 post-encephalitogen, at which point most mice were in an active disease stage of EAE, i.e., clinical disability. For both studies, mice were anesthetized with isoflurane (Abbott Labs, North Chicago, IL), dorsal surface shaved, and given two subcutaneous injections (dorsum) of the encephalitogen myelin oligodendrocyte glycoprotein peptide [amino acids 35-50; 250 μg (1^st^ study) or 300 μg (2^nd^ study)] with emulsion [Freund’s incomplete adjuvant containing 250 μg (1^st^ study) or 500 μg (2^nd^ study) *Mycobacterium tuberculosis* (Difco Laboratories, Detroit, MI)]. Once completed, an intraperitoneal (i.p.) injection of pertussis toxin (PTX; 100 ng/100 μl saline; List Biological Laboratories, Campbell, CA) was administered. Mice were given one (1^st^ study- Day 3 post-encephalitogen injection) or two (2^nd^ study- Day 3 and 7 post-encephalitogen injection) additional PTX injections. Mice were weighed on Day 0 and 7 post-encephalitogen, and every day thereafter, and scoring began on Day 9 post-encephalitogen. Mice were scored using a modified 0-8 point scale from that described previously [8]. A 0-8 point scale offers greater sensitivity to detect statistical differences between groups compared to 0-5 scales, or has a similar sensitivity to 0-5 scales that include some half point differences. Briefly, the scoring system was as follows: 0 = normal; 1 = flaccid/limp tail; 2 = hindlimb weakness causing righting difficulty from a supine position; 3 = hindlimb weakness causing righting inability ≥ 8 sec from a supine position; 4 = hindlimb weakness causing limping and abnormal gait; 5 = partial (one limb) hindlimb paralysis or extensive hindlimb weakness such that the hindlimbs cannot contribute to mobility; 6 = total (both) hindlimb paralysis plus forelimb weakness; 7 = hindlimb paralysis and forelimb weakness or paralysis resulting in a side resting position; 8 = moribund requiring sacrifice or inadvertent death. Omeprazole (Premier Pharmacy Labs, Weeki Wachee, FL) (15 mg/kg, i.p., twice daily) and saline administration began on Day 8 post-encephalitogen. Spleens were harvested in both studies for flow cytometry. Fecal pellets for bacterial analyses were collected in the 2^nd^ study on Day 40 post-encephalitogen injection. Hindbrains were immersion-fixed in 10% neutral buffered formalin (Fisher Scientific, Hanover Park, IL) and paraffin-embedded.

### EAE induction - SJL/J mice

EAE was induced in ~5-6 week old SJL/J female mice (Jackson Laboratory). Mice were anesthetized with isoflurane (Abbott Laboratories), dorsal surface shaved, and given two subcutaneous injections (dorsum) of encephalitogen [150 μg proteolipid protein peptide (amino acids 139-151)] with emulsion [Freund’s incomplete adjuvant containing 250 μg *M. tuberculosis* (Difco Laboratories)]. This was followed with an i.p. injection of PTX. Mice were also administered PTX on Day 3 post-encephalitogen injection.

Mice were weighed at Day 0 and 7 post-encephalitogen administration and every day thereafter. Clinical scoring was performed as described previously [8] except the standard for a score of 5 described above was used. Administration of omeprazole (15 mg/kg, i.p., twice daily) or saline began when a score of 1 was first detected (beginning of active disease) and continued until sacrifice. On Day 15 post-encephalitogen injection, which was a peak of disease activity, a matched subset of 5 mice within each group was sacrificed and hindbrains and spleens harvested for histopathology and flow cytometry, respectively. The remaining mice were sacrificed on Day 22 or later post-encephalitogen injection.

### Bacterial analysis

#### Sample collection & DNA extraction

On Day 40 post-encephalitogen, two freshly evacuated fecal pellets were collected per C57BL/6J mouse with EAE given omeprazole or saline. Pellets were placed into a microcentrifuge tube and immediately frozen on dry ice. Microbiome analysis was performed by the Mutant Mouse Regional Resource Center (University of Missouri-Columbia) where the pellets were transferred to 2 mL round-bottom tubes containing 800 μL lysis buffer (500 mM NaCl, 50 mM Tris-HCl, 50 mM EDTA, and 4% sodium dodecyl sulfate) and a 0.5 cm diameter stainless steel bead. Following mechanical disruption using a TissueLyser (Qiagen, Venlo, Netherlands), tubes were incubated at 70°C for 20 min with brief vortexing every 5 min. Samples were then centrifuged at 5000 × g for 5 min at room temperature, and the supernatant transferred to a clean 1.5 mL Eppendorf tube. Ammonium acetate (10 mM; 200 μL) was added to lysates, mixed thoroughly, incubated on ice for 5 min, and centrifuged at 5000 × g for 5 min at room temperature. The supernatant, 750 μL, was mixed with one volume of chilled isopropanol, mixed thoroughly, incubated on ice for 30 min, and centrifuged at 16000 × g for 15 min at 4°C. The supernatant was aspirated and discarded, and the DNA pellet was washed several times with 70% EtOH and resuspended in 150 μL of Tris-EDTA. Proteinase-K (15 μL) and Buffer AL (200 μL) (Qiagen DNeasy kit, Qiagen) were added and incubated at 70°C for 10 min, followed by addition of 200 μL of 100% EtOH. The contents of each tube were transferred to a spin column from the DNeasy kit. DNA was purified according to the manufacturer’s instructions and eluted in 200 μL of EB buffer. Purity of DNA was assessed via spectrophotometry (Nanodrop, Thermo Fisher Scientific, Waltham, MA); yield was determined via fluorometry (Qubit, Life Technologies, Carlsbad, CA) using quant-iT BR dsDNA reagent kit (Invitrogen).

#### Metagenomic library preparation and sequencing

Extracted fecal DNA was processed at the University of Missouri DNA Core Facility. Bacterial 16S ribosomal DNA amplicons were constructed by amplification of the V4 hypervariable region of the 16S rRNA with primers flanked by Illumina standard adapter sequences. Briefly, universal primers (U515F/806R), previously developed against the V4 region, were used for generating amplicons [9, 10]. Oligonucleotide sequences are available at proBase [11]. A single forward primer and reverse primers with a unique 12-base index were used in all reactions. Extracted DNA was quantitated by Qubit fluorometer using the quant-iT HS dsDNA reagent kit (Invitrogen). PCR reactions (50 μL) contained 100 ng of genomic DNA, forward and reverse primers (0.2 μM each), dNTPs (200 μM each), and Phusion High-Fidelity DNA Polymerase (1U). PCR amplification was performed as follows: 98°C^(3:00)^ + [98°C^(0:15)^ + 50°C^(0:30)^ + 72°C^(0:30)^] × 25 cycles +72°C^(7:00)^. Amplified product (5 μL) from each reaction was combined and thoroughly mixed; pooled amplicons were purified by addition of Axygen AxyPrep MagPCR Clean-up beads (50 μL) to an equal volume of 50 μL of amplicons and incubated at room temperature for 15 min. Products were washed multiple times with 80% EtOH and the dried pellet resuspended in Qiagen EB Buffer (32.5 μL), incubated at room temperature for 2 min, and then placed on the magnetic stand for 5 min. Supernatant (30 μL) was transferred to low binding microcentrifuge tube for storage. The final amplicon pool was evaluated using the Advanced Analytical Fragment Analyzer automated electrophoresis system, quantified with the Qubit fluorometer using the quant-iT HS dsDNA reagent kit (Invitrogen), and diluted according to Illumina’s standard protocol for sequencing on the MiSeq.

#### Informatics analysis

Assembly, binning, and annotation of DNA sequences were performed at the MU Informatics Research Core Facility. Briefly, contiguous sequences of DNA were assembled using FLASH software [12], and Qiime v1.7 [13] was used to select representative operational taxonomic units (OTUs). Taxonomy was assigned to selected OTUs using BLAST [14] against the Greengenes database [15] of 16S rRNA sequences and taxonomy.

### Flow cytometry

Spleens were minced using a wire mesh, and splenocytes were collected and counted. For the immunodetection of specific populations of T cells within the spleen, anti-CD4-Pacific Blue, anti-CD8-Alexa647, anti-CD24-PE, anti-CD44-PE-Cy5.5, and anti-CD62L-PE-Cy7 were purchased from BioLegend (San Diego, CA) or BD Biosciences (San Jose, CA). Cell labeling was performed in PBS containing 2% FCS. Flow cytometry studies were performed using a BD LSR II (BD Immunocytometry Systems, San Jose, CA). Data were analyzed using BD FACSDiva software (BD Biosciences). Splenocytes were gated on the live lymphocyte gate and doublet discrimination was performed. Naïve T cells were defined as CD44^lo^CD62L^hi^, central memory (CM) T cells as CD44^hi^CD62L^hi^, and effector memory (EM) T cells as CD44^hi^CD62L^lo^.

### Rotarod

The rotarod was set to accelerate from a speed of 4 to 40 rotations per minute in a 300 second time trial. Each mouse was given an exposure trial to familiarize the animal to the task, and this initial trial was not included for data analysis. Each animal was then given two trials and the times at which mice could no longer successfully manage or remain on the rotarod (rotarod latency) were averaged and analyzed for differences between treatment groups and relationships to EAE clinical scores.

### Histopathology

Following paraffin embedding, sagittal sections of the hindbrain were cut at 8 μm thickness and processed for hematoxylin and eosin staining. Sections were evaluated on the basis of number of lesions and magnitude of lesions present in the hindbrain.

### Statistics

The two-tailed Student’s t-test was used to evaluate weight change, difference in absolute numbers of subsets of spleen cells, and rotarod latency between omeprazole and saline groups. The Wilcoxon two sample test was employed to evaluate percent differences in bacterial microbiota, percent CD4^+^ and CD8^+^ spleen cells, max clinical scores, and area under the curve (sum clinical scores) between groups. Statistical significance was set at p ≤ 0.05 for both the Student’s t-test and the Wilcoxon two sample test. For regression analysis between clinical score and rotarod latency, the r^2^ value and statistical significance (p ≤ 0.05) were evaluated using GraphPad Prism 6 software (GraphPad Software, Inc., San Diego, CA).

## Results

### Omeprazole affects the gut microbiota in C57BL/6J mice with EAE

**Figure 1:**
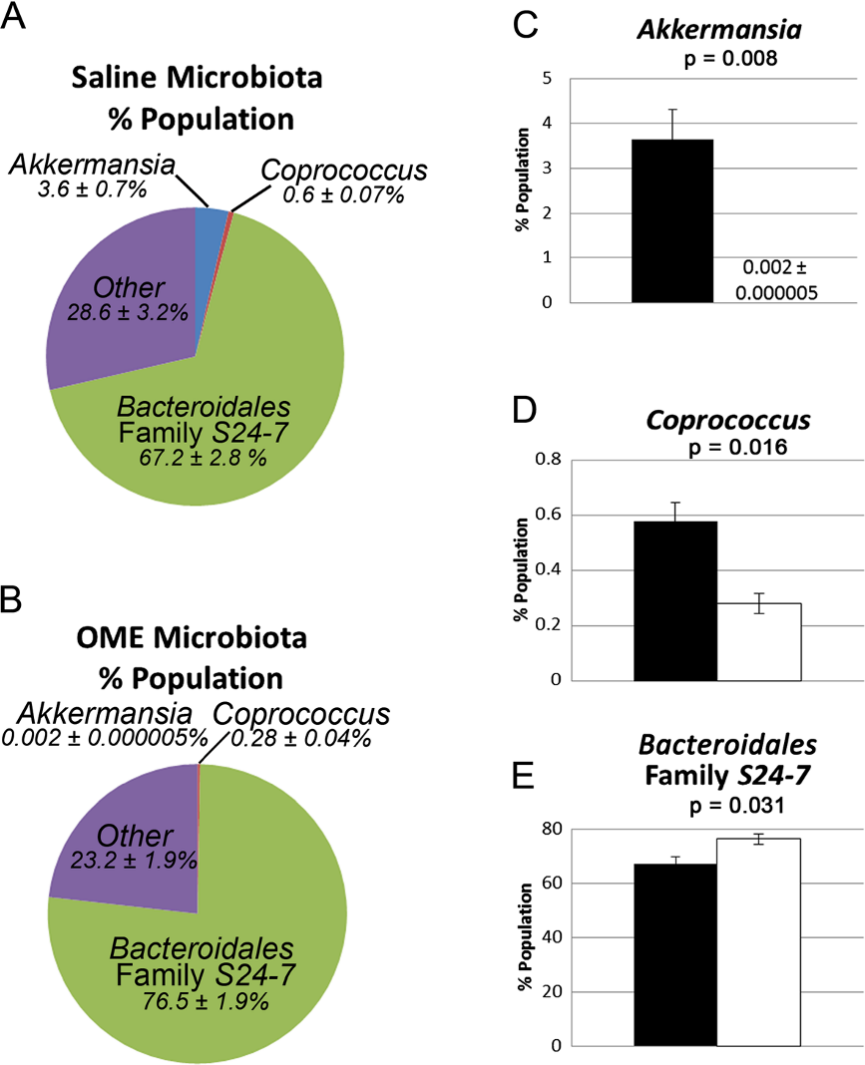
**Next-generation sequencing of fecal microbiota from omeprazole and saline treated C57BL/6J mice.** Pellets were collected on Day 40 post-encephalitogen during active disease from a subset of omeprazole (n = 5) and saline (n = 5) treated C57BL/6J mice. Sequences were annotated against a database of known 16S rRNA gene sequences and binned at all taxonomic levels (phylum, class, order, etc.). Operational taxonomic units (OTUs) represented in these graphs were statistically different following omeprazole treatment in C57BL/6J mice. Overall distribution in saline **(A)** and omeprazole **(B)** treated mice and more detailed analyses of three different OTUs **(C-E)** are shown. Black bars - saline treatment; white bars - omeprazole treatment. Additional information is provided in Additional file 1: Table S1.

The gut microflora has been shown to affect EAE disease progression [4–6]. Since omeprazole can elevate the gastric pH [16], which could affect the growth of populations of bacteria in the gut [17], omeprazole has the potential to affect the progression of EAE. We first sought to determine the effect of chronic omeprazole treatment on the bacterial gut microbiota in C57BL/6J mice with EAE. Shifts in percentages of specific bacteria populations were identified following omeprazole treatment (Figure 1A, B); in particular, there were significant decreases in *Akkermansia muciniphila* and *Coprococcus* sp. and an increase in unidentified microbes in the family S24-7 (order *Bacteroidales*), which was the most abundant OTU in the fecal samples (Figure 1C-E; Additional file 1: Table S1). There were numerous different bacterial taxa detected (Figure 1A, B; Additional file 1: Table S1), but there was no significant difference in their relative abundance between the omeprazole and saline EAE mice, or they represented < 0.1% of the total bacterial population.

### Omeprazole does not affect spleen T cell content in C57BL/6J mice with EAE

**Figure 2:**
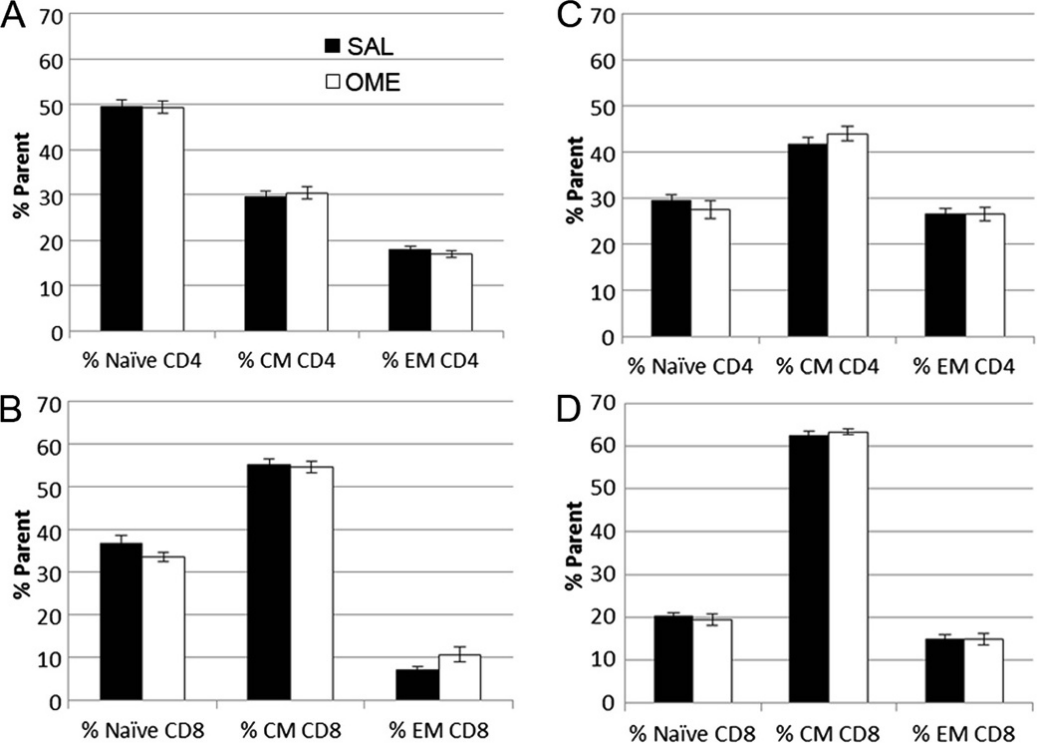
**Evaluation of spleen immune cell content in omeprazole and saline treated C57BL/6J mice.** Quantitation of spleen immune cells from flow cytometry analysis in saline and omeprazole treated C57BL/6J mice was performed on Day 18 post-encephalitogen **(A, B)**, which was at an early clinical stage (clinical scores: saline 2.36 +/- 0.84, n = 7 vs. omeprazole 1.21. +/- 0.15, n = 7; p = 0.15) and Day 46 post-encephalitogen **(C, D)**, which was during an advanced disease stage (clinical scores: saline 4.14 +/- 0.7, n = 7 vs. omeprazole 4.14 +/- 0.14, n = 7; p = 1.0). No differences in the percent of naïve (CD44^lo^CD62L^hi^), CM (CD44^hi^CD62L^hi^) or EM (CD44^hi^CD62L^lo^) CD4+ or CD8+ cells (from parent CD4+ or CD8+ populations) were observed following omeprazole treatment vs. saline during both stages of EAE. Black bars, saline treatment; white bars, omeprazole treatment.

Since the spleen is a key site of T cell activation and has a prominent role in the development of EAE [18], the effect of omeprazole on spleen T cell populations was examined. T cells collected at an early clinical stage (Day 18) or during an active disease stage (Day 46) displayed no differences between omeprazole and vehicle administration (Figure 2A-D). However, there was a difference in the spleen immune cell populations between these different stages of disease progression. There were statistically significant higher numbers of EM CD4^+^ T cells, CM CD4^+^ T cells, EM CD8^+^ T cells, and CM CD8^+^ T cells, and lower numbers of naïve CD4^+^ T cell and CD8^+^ T cells in the advanced disease stage compared to the early clinical stage (Additional file 2: Figure S1A).

### Omeprazole does not affect clinical or pathological signs in C57BL/6J mice with EAE

**Figure 3:**
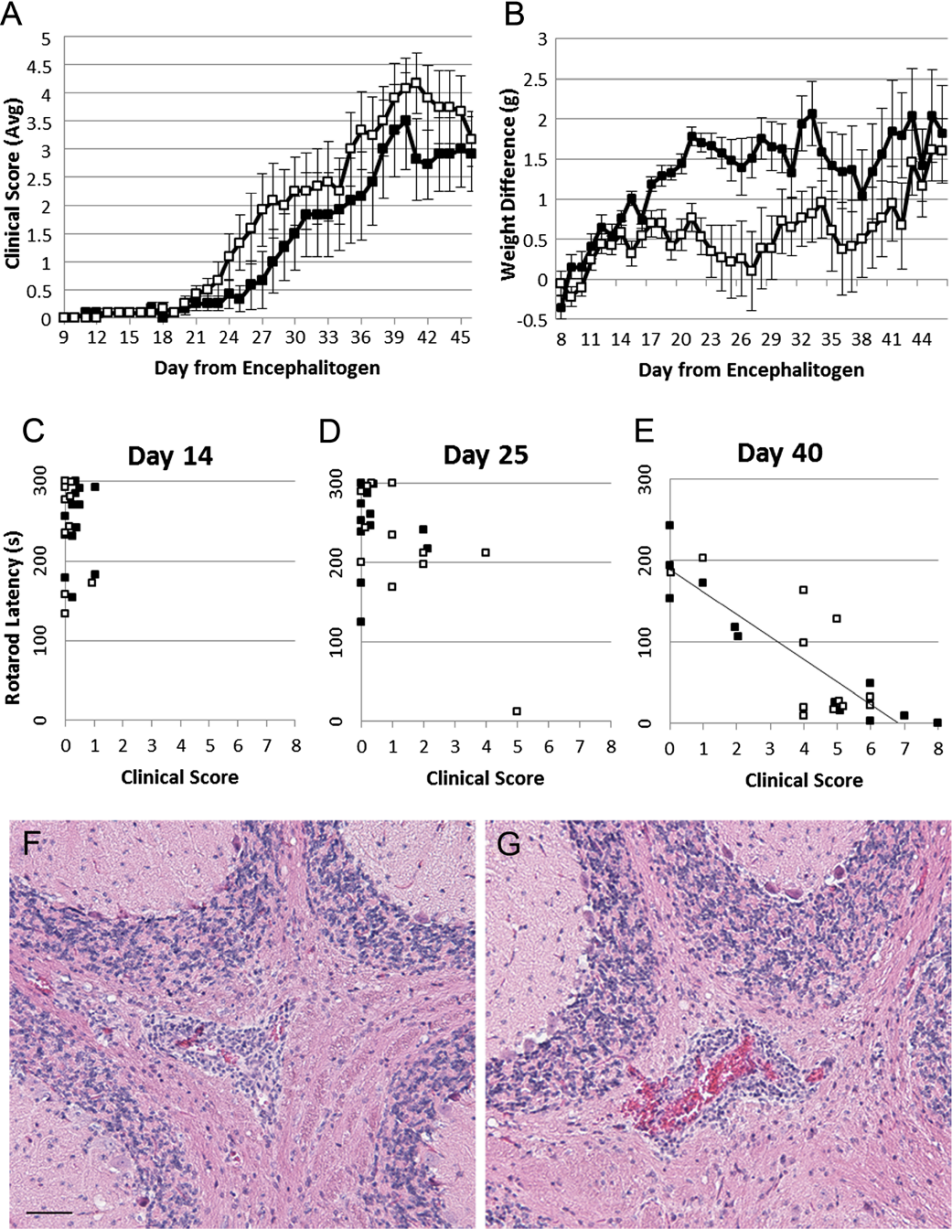
**Comparison of omeprazole and saline treated C57BL/6J mice with EAE.** Average clinical scores **(A)** revealed no significant differences between groups, but weight differences **(B)** revealed that omeprazole treated EAE mice (n = 12) had significantly less weight gain relative to Day 7 than saline EAE mice (n = 12). Rotarod latency at Day 14 **(C)**, Day 25 **(D)**, and Day 40 **(E)** post-encephalitogen, with no differences observed between omeprazole and saline groups. A strong negative correlation was observed between clinical score and rotarod latency during active disease at Day 40 post-encephalitogen (r^2^ = -0.747, p < 0.005), but not at Day 14 or Day 25. Black squares, saline treatment; white squares, omeprazole treatment. Histological examination revealed no major differences in inflammatory cell infiltrates in the hindbrains of both saline **(F)** and omeprazole **(G)** C57BL/6J mice during active disease. Bar = 50 μm.

The first detection of EAE disease activity in most C57BL/6J mice was approximately 18 days or greater post-encephalitogen injection, about the time at which they were ~7 ½ weeks old, although occasionally a low percentage of mice became sick earlier. This delayed onset compared to other studies was likely due to injection of the encephalitogen into ~5 week old C57BL/6J mice rather than older C57BL/6J mice used in other studies [6, 19]. After disease onset, the progression of clinical scores appeared to advance more rapidly in the EAE mice administered omeprazole compared to saline, but the differences did not achieve significance (Figure 3A). However, the differences in weight were significant (less weight gain) in the omeprazole group vs. the saline group (Figure 3B). No differences were observed in rotarod latency between groups receiving omeprazole and saline administration during any stage of EAE disease progression (Day 14, 25 or 40 post-encephalitogen injection) (Figure 3C-E), and thus, data were pooled for comparisons of rotarod latency and clinical scores. Comparison of the relationship between rotarod latency and clinical scores revealed that at Day 14 and Day 25 the two clinical measures did not correlate well (Figure 3C, D); but by Day 40, there was a strong correlation between rotarod latency and clinical scores (r^2^ = 0.747, p < 0.005; Figure 3E). Inflammatory cell infiltrates were present in the hindbrains of both omeprazole and saline treated mice during active disease, but there was no major difference between treatment groups (Figure 3F, G).

### Omeprazole does not affect EAE disease activity in SJL/J mice

**Figure 4:**
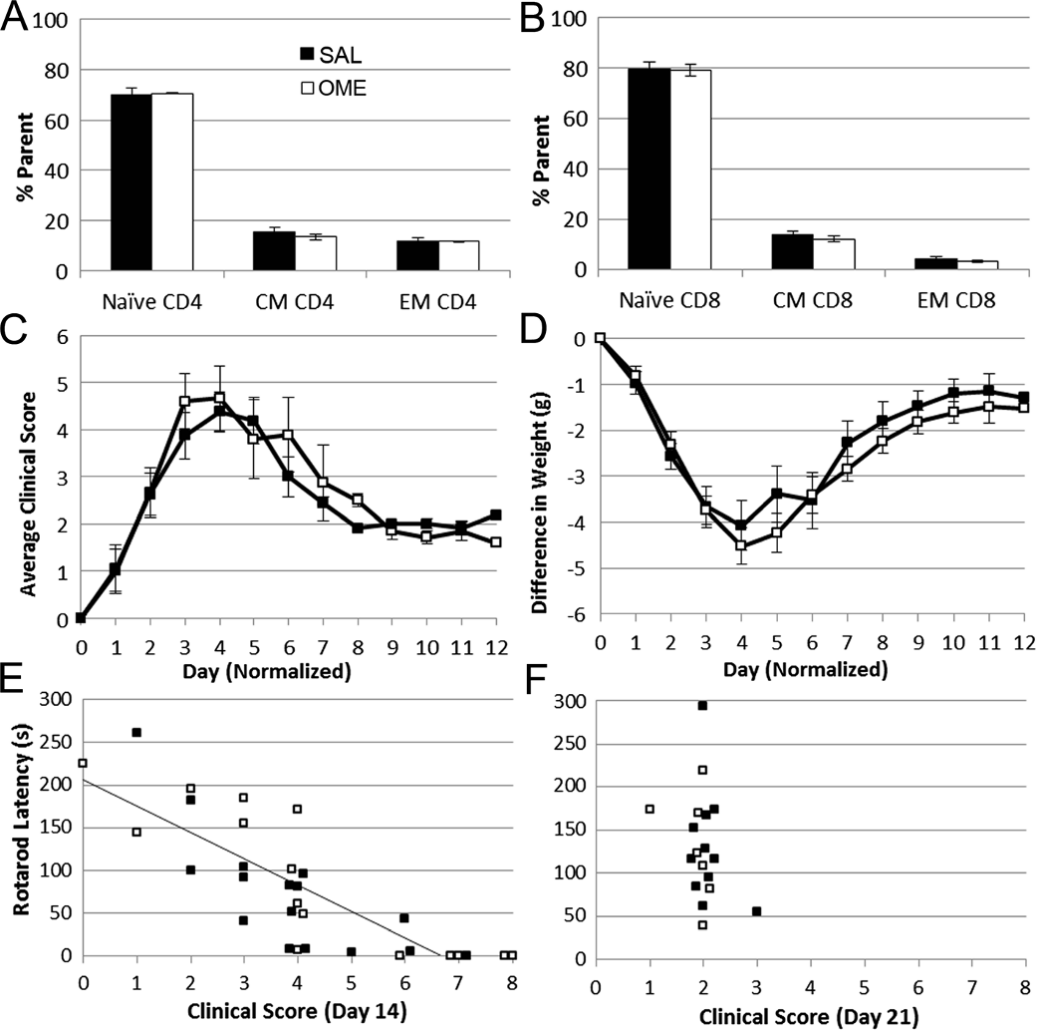
**Evaluation of omeprazole and saline treated SJL/J mice with EAE.** Quantitation of percent of naïve, CM or EM CD4+ or CD8+ cells (relative to respective parent CD4+ or CD8+ populations) was performed on spleen cells from saline (n = 5) and omeprazole (n = 5) treated SJL/J mice during active disease **(A, B)**. No significant differences were observed following omeprazole treatment compared to saline in the relative percentages of all T cell populations examined. Average clinical scores **(C)** and weight difference from the day prior to disease onset **(D)** in saline and omeprazole treated SJL/J mice. No significant differences were observed between groups (n = 15 for the omeprazole group and n = 16 for the saline group at the start of the study, with 5 mice per group sacrificed at Day 15 and used for splenocyte analysis, above). Rotarod latency plotted vs. clinical scores during active disease, Day 14 post-encephalitogen **(E)** and remission, Day 21 post-encephalitogen **(F)**. No differences were observed following omeprazole treatment vs. saline at either disease stage. A negative correlation was observed between clinical score and rotarod latency on Day 14 post-encephalitogen (r^2^ = -0.653, p < 0.005).

On average, EAE in SJL/J mice was first detected by ~10 days post-encephalitogen. Administration of omeprazole or saline was started at the initiation of clinical signs (Day 0 of treatment). Examination of the immune cell content in spleens during active disease revealed that administration of omeprazole did not alter the T cell content compared to saline (Figure 4A, B). Furthermore, there was no difference in the clinical disease profile between omeprazole and saline administered mice with EAE, i.e., both groups developed advanced disease (e.g., clinical score of 6 or 7 and substantial weight loss) which was followed by remission (e.g., clinical scores dropping to ~2 associated with weight gain) (Figure 4C, D). There was also no significant difference in rotarod behavior between mice administered omeprazole or saline during active disease or remission (Figure 4E, F), and thus the data were pooled between groups for comparisons of rotarod latency and clinical scores. There was a strong correlation between clinical score and rotarod latency during active disease (r^2^ = 0.653, p < 0.005; Figure 4E), but not during remission (Figure 4F) in SJL/J mice, at which point a heterogeneity in rotarod latency was revealed despite most mice displaying a similar clinical score of 2 (Figure 4F). Both omeprazole and saline treated EAE mice displayed an abundance of inflammatory cell infiltrates in the hindbrain (data not shown).

Comparisons of the spleen T cell content between the two strains of mice with active EAE revealed that total and naïve CD4^+^ and CD8^+^ cells were significantly higher in SJL/J mice than in C57BL/6J mice, and there were more CM CD4^+^ and CD8^+^ T cells in the C57BL/6J EAE mice compared to SJL/J EAE mice (Additional file 2: Figure S1B).

## Discussion

The gut microflora can affect EAE disease progression [4–6]. For instance, segmented filamentous bacteria [6], likely from the genus *Clostridium* [20], were found to augment EAE while *Bacteroides fragilis* limits EAE development [21]; and depending on the species of *Lactobacillus*, EAE disease activity can be increased [22] or ameliorated [23]. In our study, *Clostridium* was not significantly different between treatments (0.18 ± 0.13 saline; 0.22 ± 0.11 omeprazole), and *Bacteroides fragilis* and *Lactobacillus* sp. were below 0.1% in both groups (saline or omeprazole) of EAE mice. Thus, omeprazole treatment did not appear to substantially affect gut bacteria currently known to influence EAE development.

*Akkermansia muciniphila* was significantly lower in the omeprazole treated EAE mice compared to vehicle treated EAE mice. *Akkermansia muciniphila* is a mucolytic bacterium that is found in the mucus layer of the large intestine [24]. Omeprazole has been shown to inhibit mucin production by the stomach [25, 26]. Thus, a lower production of mucins following omeprazole treatment likely made it difficult for *Akkermansia muciniphila* to thrive, which could account for the significantly diminished levels of *Akkermansia muciniphila* following omeprazole treatment. Low levels of *Akkermansia muciniphila* have been associated with obesity and type 2 diabetes in C57BL/6J mice [27] *.* However, in the present study, EAE mice given omeprazole had less weight gain, despite a lower percentage of *Akkermansia muciniphila*, compared to EAE mice given saline. The findings with the unidentified OTU in order *Bacteroidales*, family S24-7 might better explain the weight changes.

The relative abundance of this OTU (order *Bacteroidales*, family S24-7) increased in the present study following omeprazole treatment. An increased level of this taxon has been observed following exercise [28] and in lean mice compared to obese mice [29]. Our study did not measure absolute levels of bacteria, but rather evaluated the relative abundance of different types of bacteria. However, bacterial overgrowth can occur in the stomach and duodenum following omeprazole treatment [17], and bacterial growth and gastric acid suppressors have both been associated with weight loss in the elderly [30]. Thus, it may not be surprising that omeprazole treated EAE mice had less weight gain than saline treated EAE mice.

The percentage of *Coprococcus* sp. decreased following omeprazole treatment. Little is known about the genus *Coprococcus* relative to EAE. Its abundance is increased in Crohn’s disease [31] and is decreased following exposure to social disruption stress [32] or in HIV individuals not taking combination anti-retroviral therapy [33].

Besides affecting the gut microbiota, PPIs have been shown to affect neutrophil function, which recently has been implicated to play a role in disease initiation in EAE [34, 35]. In particular, omeprazole can decrease the migration, bactericidal activity, and oxygen-derived free radical production by neutrophils [36–38]. PPIs can also affect NK cells [39] and monocytes [40]. However, MS and EAE are primarily T cell driven diseases [41–47]. It is unknown whether omeprazole directly affects T cells, but the PPI esomeprazole was thought to create a microenvironment, via a change in pH, more suitable for T cell activity against tumors [48]. In the present study, omeprazole treatment did not affect levels of different subsets of T cells (e.g., CM, EM) in the spleen of C57BL/6J or SJL/J EAE mice. Furthermore, omeprazole did not affect relative levels of inflammatory cell infiltrates in the hindbrain, which are largely composed of T cells, in either strain, although it is possible that omeprazole could affect other components of the T cell response.

Aside from differences in weight gain in C57BL/6J EAE mice, omeprazole did not affect other clinical measures of disease activity, i.e., clinical scores and rotarod latency, in either C57BL/6J or SJL/J EAE mice. Independent of treatment, rotarod latency did not appear to correlate with clinical scores during the preclinical or early stage of EAE in C57BL/6J mice, but a strong correlation was present in animals with active disease, and this correlation was also observed for SJL/J mice with active disease. However, once the SJL/J mice entered into remission, based on clinical signs, the correlation was lost. For example, almost all the SJL/J mice had a clinical score of 2 during remission, yet there was a wide heterogeneity in rotarod latency values which did not appear to correlate to a prior maximum clinical score or with hindbrain pathology (i.e., inflammatory cell infiltrates). Thus, rotarod testing revealed that mice in remission had a range of persistent deficiencies that were not reflected in the clinical score. Therefore, we suggest that rotarod latency can be a useful measure to complement clinical scoring for disease assessment, particularly during later stages of disease. Interestingly, the expanded disability status scale has quantitative measures of mobility included in the evaluation of disease status in MS patients [49]; thus, including both clinical scores and rotarod latency for measurement of EAE activity would more closely align the evaluations used for human and rodent studies.

Th1 CD4^+^, CD8^+^ and/or Th17 T cells are important contributors to disease activity in EAE and MS [41–47]. The spleen T cell population changed during different stages of disease in C57BL/6J mice. A decrease of naïve CD4^+^ and CD8^+^ cells was associated with an increase in both EM and CM CD4^+^ and CD8^+^ cells during active disease compared to early disease. Furthermore, the total number of CD4^+^ and CD8^+^ spleen cells were increased during active disease. These changes likely reflect the heightened activation of the immune system during active disease and support the finding that effector and memory T cells affect the development of EAE [50].

During active disease, a higher percentage of naïve CD4^+^ and CD8^+^ and a lower percentage of CM CD4^+^ and CD8^+^ cells and EM CD8^+^ cells were observed in SJL/J mice compared to C57BL/6J mice. There was also a difference in the absolute cell counts in the spleen of the two strains, with SJL/J having nearly twice as many cells as the C57BL/6J. These differences are likely accounted for by apoptosis and spleen atrophy, which have been observed in spleens of mice with progressive EAE, but not with relapsing-remitting EAE [51].

An observation during the procedures used for this study was that defecation appeared less frequent in EAE mice with advanced disease compared to healthier mice, regardless of treatment. This may have been due to a difference in food consumption or a disruption of nervous control over peristalsis and/or defecation as EAE progressed; of note, bowel dysfunction occurs in a large percentage of MS patients, which can be due to a variety of causes [52].

## Conclusions

Although there was a difference in the microbiota and weight gain in the C57BL/6J EAE model, omeprazole had no effect on spleen T cell populations, clinical scores, rotarod, and histopathology in C57BL/6J and SJL/J EAE mice. Thus, omeprazole does not appear to have a positive or negative effect on the disease course of EAE, suggesting that omeprazole may not be affecting disease activity in MS patients. However, despite testing in two EAE models, the design used in the present study has limitations relative to applicability to humans. Besides the obvious limitation of extrapolating results from mice with EAE to humans with MS, the gut microbiota in each individual is a result of multiple factors; thus, it is theoretically possible that in specific situations a PPI could cause a shift in the microbiota, or other factor, that influences MS disease activity. As we learn more about the influences of the gut microflora on MS disease activity, it might become relevant to revisit the role of PPIs in MS.

## Electronic supplementary material

Additional file 1: Table S1: Operational taxonomic units. (XLSX 28 KB)

Additional file 2: Figure S1: Since there were no clear differences between saline and omeprazole treatment in the percent of naïve, CM or EM CD4+ or CD8+ cells (from parent CD4+ or CD8+ populations) (Figure 2), mice from the omeprazole and saline groups were pooled and used to analyze the differences in immune cell populations between the two different stages of EAE in C57BL/6J mice (clinical scores at Day 18 vs. Day 46, p = 0.0009) using absolute numbers of the total spleen cell population **(A)**. More CM and EM CD4+ and CD8+ were observed at the advanced disease stage and more naïve CD4+ and CD8+ cells were observed during the early clinical stage (* p < 0.05; ** p < 0.001; *** p < 0.00001). Comparison of spleen cells from SJL/J mice with active disease (Day 15) and C57BL/6J mice with active disease (Day 46) **(B)**. Since there were no clear differences between saline and omeprazole treatment, mice from the omeprazole and saline groups were pooled to analyze the differences in immune cell populations between the two different strains of EAE using absolute numbers of the total spleen cell population. More CM and EM CD8+ cells and more CM CD4+ cells were observed in C57BL/6J mice compared to SJL/J mice with active disease (* p < 0.01; ** p < 0.000001). Conversely, fewer total and naïve CD4+ and CD8+ cells were observed in C57BL/6J mice compared to SJL/J mice with active disease (** p < 0.000001). Black bars or squares, saline treatment; white bars or squares, omeprazole treatment. (TIFF 361 KB)

## Abbreviations

CM: Central memory
EAE: Experimental autoimmune encephalomyelitis
EM: Effector memory
i.p.: Intraperitoneal
MS: Multiple sclerosis
OTUs: Operational taxonomic units
PTX: Pertussis toxin
PPI: Proton pump inhibitors.
