# Supplementary figures and images for: The effect of omeprazole on the development of experimental autoimmune encephalomyelitis in C57BL/6J and SJL/J mice
Source: BMC Res Notes. 2014 Sep 4;7:605. doi: 10.1186/1756-0500-7-605 (PMC4167283; doi:10.1186/1756-0500-7-605)

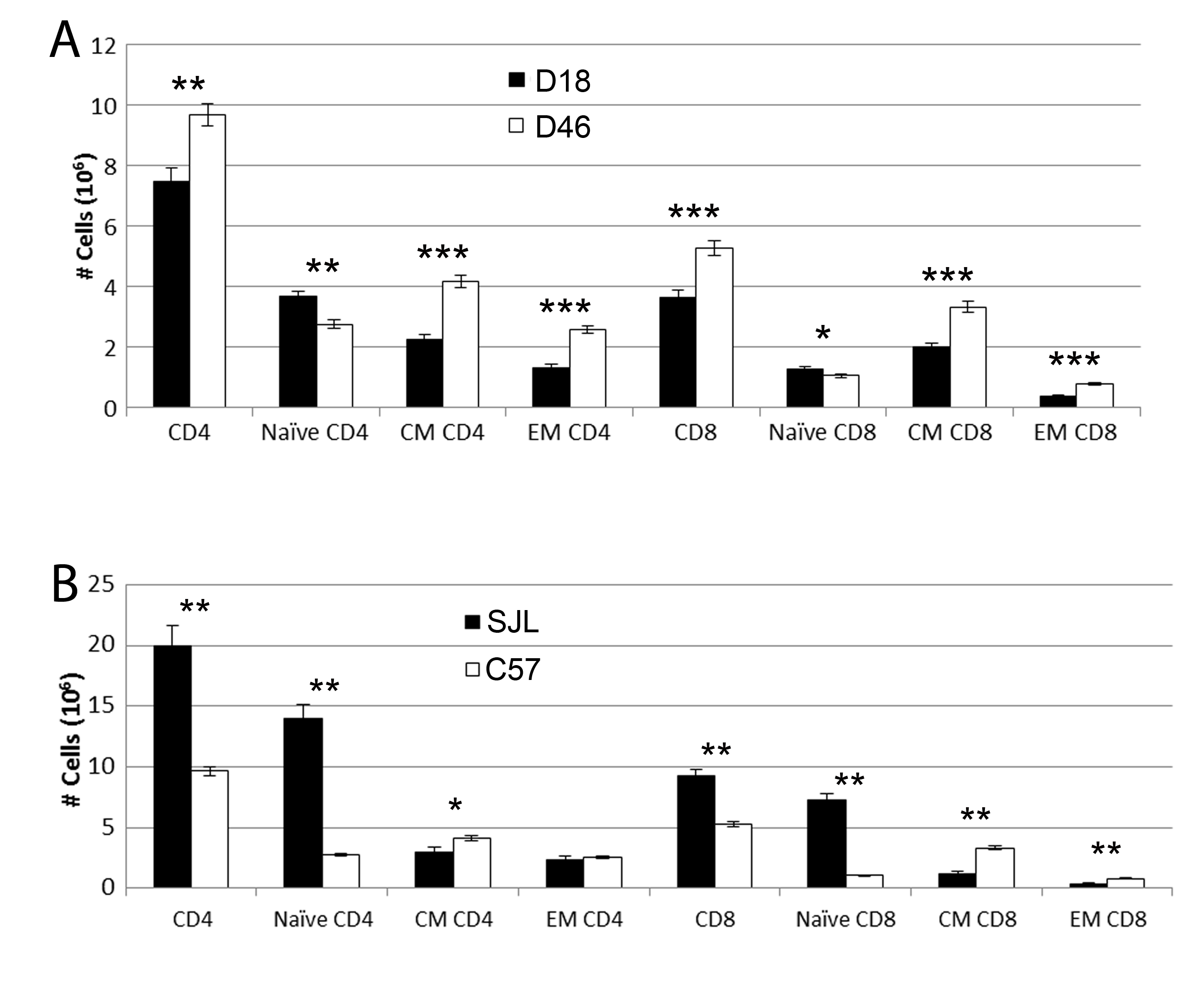

Supplement: Supplementary file 2 — Additional file 2: Figure S1: Since there were no clear differences between saline and omeprazole treatment in the percent of naïve, CM or EM CD4+ or CD8+ cells (from parent CD4+ or CD8+ populations) (Figure 2), mice from the omeprazole and saline groups were pooled and used to analyze the differences in immune cell populations between the two different stages of EAE in C57BL/6J mice (clinical scores at Day 18 vs. Day 46, p = 0.0009) using absolute numbers of the total spleen cell population (A). More CM and EM CD4+ and CD8+ were observed at the advanced disease stage and more naïve CD4+ and CD8+ cells were observed during the early clinical stage (* p < 0.05; ** p < 0.001; *** p < 0.00001). Comparison of spleen cells from SJL/J mice with active disease (Day 15) and C57BL/6J mice with active disease (Day 46) (B). Since there were no clear differences between saline and omeprazole treatment, mice from the omeprazole and saline groups were pooled to analyze the differences in immune cell populations between the two different strains of EAE using absolute numbers of the total spleen cell population. More CM and EM CD8+ cells and more CM CD4+ cells were observed in C57BL/6J mice compared to SJL/J mice with active disease (* p < 0.01; ** p < 0.000001). Conversely, fewer total and naïve CD4+ and CD8+ cells were observed in C57BL/6J mice compared to SJL/J mice with active disease (** p < 0.000001). Black bars or squares, saline treatment; white bars or squares, omeprazole treatment. (TIFF 361 KB) [file 13104_2014_3135_MOESM2_ESM.tiff]
